# No Evidence for Temperature-Dependence of the COVID-19 Epidemic

**DOI:** 10.3389/fpubh.2020.00436

**Published:** 2020-08-26

**Authors:** Tahira Jamil, Intikhab Alam, Takashi Gojobori, Carlos M. Duarte

**Affiliations:** ^1^Computational Bioscience Research Center (CBRC), King Abdullah University of Science and Technology, Thuwal, Saudi Arabia; ^2^Red Sea Research Centre (RSRC), King Abdullah University of Science and Technology, Thuwal, Saudi Arabia

**Keywords:** COVID-19, epidemic, temperature, exponential rate, Rt

## Abstract

The pandemic of the COVID-19 extended from China across the north-temperate zone, and more recently to the tropics and southern hemisphere. The hypothesis that COVID-19 spread is temperature-dependent was tested based on data derived from nations across the world and provinces in China. No evidence of a pattern between spread rates and ambient temperature was found, suggesting that the SARS-CoV-2 is unlikely to behave as a seasonal respiratory virus.

## Introduction

On 30th January the WHO declared the novel coronavirus (COVID-19) outbreak a public health emergency of international concern (http://www.euro.who.int/en/home). The epidemic spread gradually from Wuhan province in China, to other Asian nations, the middle east and Europe. By early March the epidemic was mostly concentrated in territories extending between 30 and 50° N (1), now in late winter, leading to the suggestion, echoed by the global media, that the epidemic is likely to be temperature-dependent. This supported speculation of possible decline in severity with the onset of warmer spring and summer temperatures in north-temperate latitudes (1, 2), comparable to many viruses affecting human respiratory systems, including SARS (3, 4).

However, recent (updated up to May 31, 2020; cf. Methods) data revealed the spread of the epidemic across territories experiencing warm temperatures in the tropics (e.g., Indonesia, Singapore, Brazil) and southern hemisphere as well (e.g., Australia, Argentina). The current distribution of the epidemic challenges, therefore, the inference that SARS-CoV-2 may behave as a seasonal respiratory virus based on previous statistical analyses from earlier realized distributions.

Here we examine the relationship between the apparent exponential rate of SARS-CoV-2 spread (γ) and the Effective Reproductive number (Rt) of infection and the average daily temperature (T_avg_) across nations and Chinese provinces where epidemics, with at least 1,000 cases reported, have been reported (data updated up to 31 May, 2020).

## Methods

### Novel Coronavirus (COVID-19) Cases Data

The Novel Coronavirus (COVID-19) daily data are confirmed cases for affected countries and provinces of China reported between 31st December 2019 and 31st May 2020. The data was collected from the reports released by WHO, European Centre for Disease Prevention and Control (ECDC), and John Hopkin CSSA. Data include confirmed and a cumulative total of COVID-19 cases in affected countries/provinces.

### Average Ambient Temperature

The average temperatures of all the affected countries were collected from https://www.timeanddate.com/weather/. The monthly mean temperature of February to May 2020 of capital cities for the various nations were used as reference temperatures for the country.

### Statistical Analysis

To avoid the estimates to be biased by confinement measurements rather than displaying the epidemic spread, we calculated the exponential rate and Effective reproduction number (Rt) of epidemic spread for the period of exponential growth in number of cases, which was defined as that showing a linear slope when plotting number of cases vs. time. We analyzed the data where the COVID-19 incident has at least a 10-day growth period, and the total number of cases was at least 1,000.

Hence, we fitted the exponential model to each country and each province of China and calculated exponential rate parameters for the countries

$$\begin{array}{l} N=ae^{\gamma Days},\;\gamma >0\\ logN=\alpha +\gamma \;Days. \end{array}$$

Where N is the cumulative number of diagnosed persons and Days is the number of days and γ is the exponential rate (100 x γ = % increase per day).

To calculate the effect of temperature on the exponential rate parameter, we first regressed the exponential rate parameters retrieved from the exponential model on *T*_*avg*_ and $T_{avg}^{2}$

$$\begin{array}{l} \gamma \sim T_{avg}+T_{avg}^{2} \end{array}$$

If the squared term is significant, it provides evidence of non-linearity.

The thermal performance of COVID-19 was characterized by fitting spread rate estimate or growth parameter (γ) and temperature to the Gaussian function;

$$\begin{array}{l} \gamma =ae^{\left[-0.5{\left(\frac{\left(T_{avg}-opt\right)}{tol}\right)}^{2}\right]}, \end{array}$$

*T*_*avg*_ is the average temperature (in °C) that best encompasses the growth period of COVID-19 cases since its 100 incidences in a country/region of China. Where, amplitude (*a*) is the coefficient related to maximum of spread rate of countries, the optimum (*opt*) on the temperature gradient is where the maximum of spread rate is attained and the tolerance (*tol*) gives the width of the response curve. This model has non-linear form, and the model parameters *opt* and *tol* occur non-linearly in the model function. Parameter of thermal performance curve was estimated by fitting Gaussian model to the growth rate and temperature of infected countries. The initial values for the Gaussian parameters *opt*, *tol*, and *a* were obtained directly using maximum-likelihood polynomial regression for the Gaussian function.

Estimated the Effective reproductive number (Rt), the average number of infections at time t, per infected case over the course of their infection for COVID-19 for provinces of China and other countries using a discrete γ distribution with a mean of 4.8 days and a standard deviation of 3.5 days for the *serial interval* distribution.

All analyses were performed using R statistical computing software. The data set is available from Jamil et al. (5).

## Results

Our results show that evidence for a temperature-dependence of the transmission reported in previous papers was likely to be spurious, reflecting the pathways of spread, and that there is no evidence for thermal dependence of the transmission across the 1–34°C T_avg_ range across the affected regions. This suggests little basis to expect evidence for the virus to behave as a seasonal respiratory virus.

**Figure 1:**
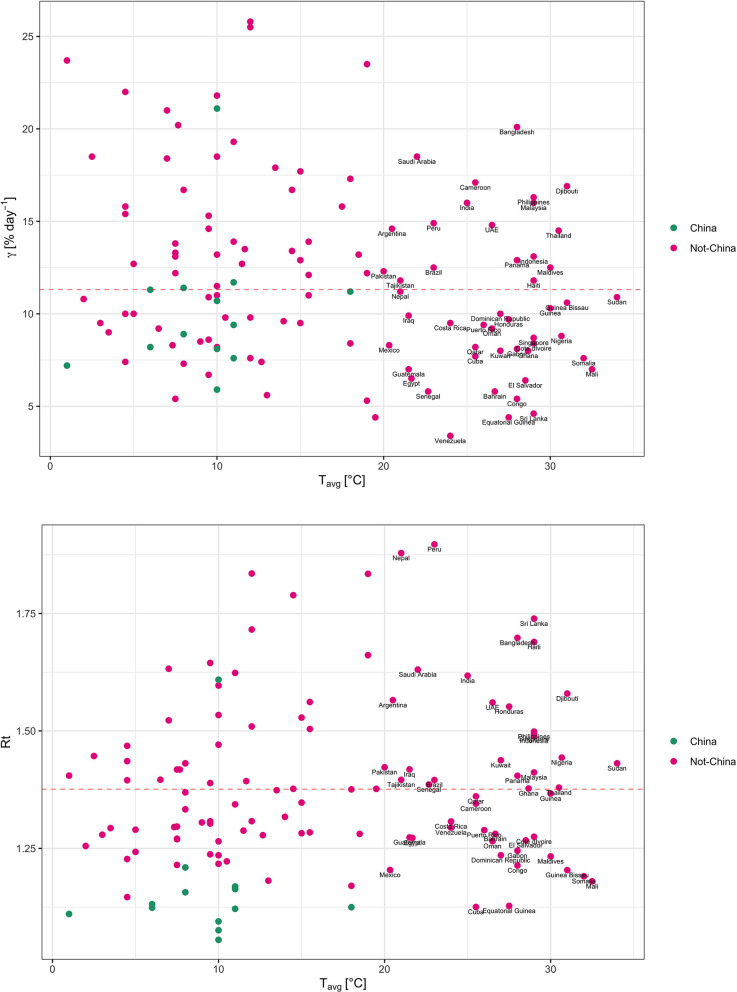
The relationship between the apparent exponential rate of SARS-CoV-2 spread (γ) and the Effective Reproductive number (Rt) and the average daily temperature (T_avg_) across nations and Chinese provinces for the period of exponential growth in number of cases of COVID-19 (data last accessed June 1, 2020, Figure S1). Green symbols represent provinces in China while red symbols represent other nations.

Epidemiological data consisting in the rate of increase in accumulated diagnosed cases among nations (global) shows γ ranging from 3.4% day^−1^ to 25.8% day^−1^ (Figure S1), with an average of 12.06 ± 0.45 % day^−1^ (Figure 1, Figure S1), and apparent Rt of 1.4 ± 0.02 (Figure 1). Surprisingly, γ and Rt across Chinese provinces (mean ± SE = 10.17 ± 0.96 % day^−1^ and 1.17 ± 0.04) were below those of other nations (mean ± SE = 12.06 ± 0.45 % day^−1^ and 1.4 ± 0.02), possibly because much faster confinement of the Chinese population did not allow for the potential exponential rates under uncontrolled conditions to be realized. The broad variability in realized γ and Rt between nations (global) and provinces (China) largely reflects differences in detection likelihood along with the timing and rigor of adoption of confinement measures.

**Figure 2:**
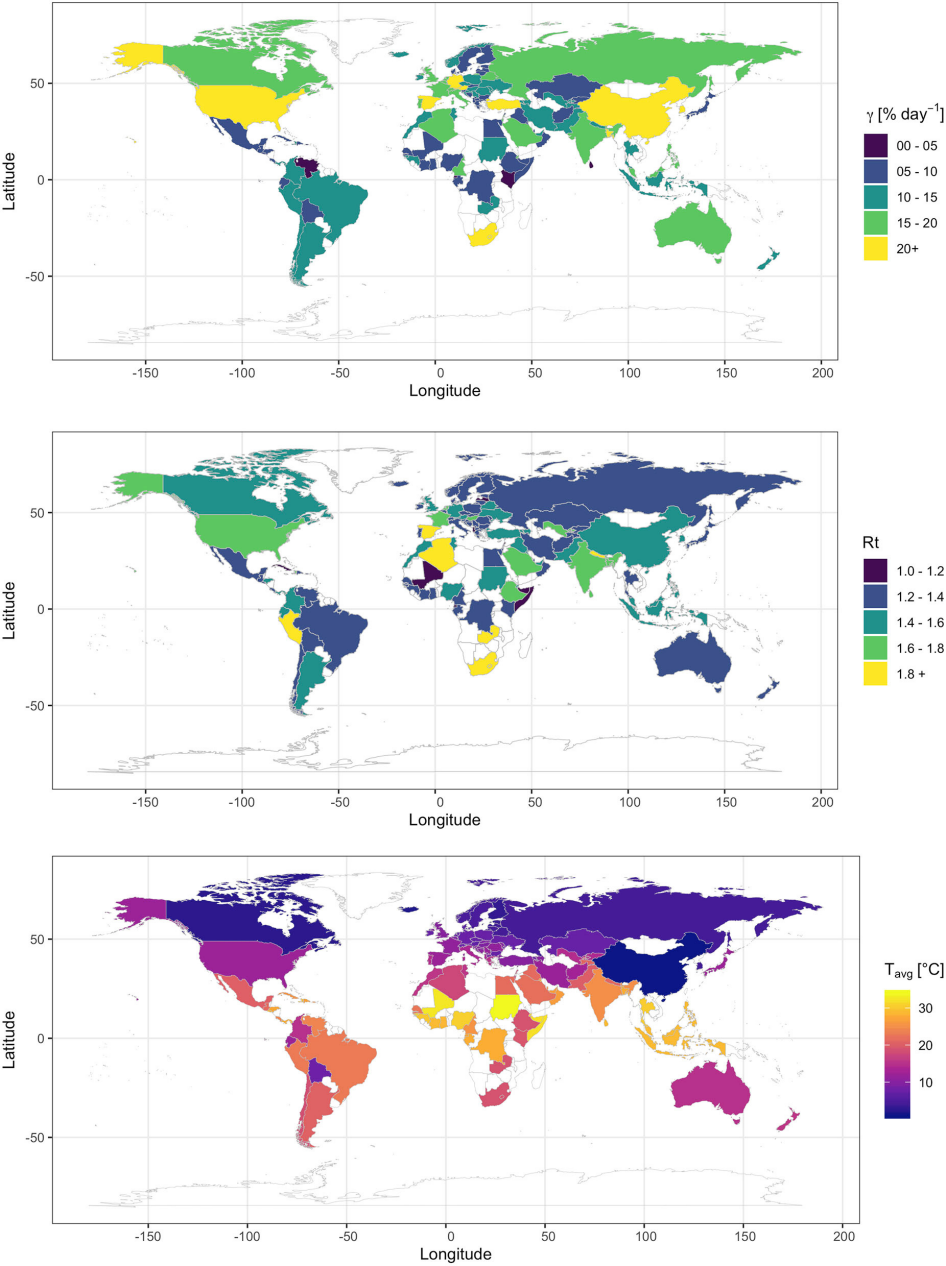
Spatial distribution of the apparent exponential rate of SARS-CoV-2 spread (γ) and the Effective Reproductive number (Rt) and the average daily temperature (T_avg_) across nations (data last accessed June 1, 2020).

The relationship between γ and Rt and T_avg_ shows no evidence for a reduced spread rate with warming (Figure 1), unlike analyses based on previous data. A number of nations with T_avg_ > 20°C, including subtropical and tropical (Brazil, Qatar, Saudi Arabia, UAE, India, and Indonesia), and southern-hemisphere (Peru, Chile, Argentina) nations (Figure 2), support γ and Rt above the median values of 11.3% day^−1^ and 1.38, respectively (Figure 1). However, the same analysis conducted on earlier data of 15th March, did provide some evidence for low γ and Rt for T_avg_ > 20°C (Figure S2). Our updated results (Figure 1) and same analysis conducted on 27th March and 31st May (Figures S3, S4) show, however, that this apparent temperature-dependence was confounded with a prevailing zonal pattern of spread across the north-temperate zone, possibly reflecting the main patterns of human mobility, which delayed arrival of the epidemics to the southern hemisphere and the tropics.

## Discussion

The results suggest that, contrary to prior assessments, the spread rate of the COVID-19 pandemic is temperature-independent, suggesting that there is little hope for relief as temperatures in the northern hemisphere increase, and that poor nations with weak health systems in tropical regions, such as African, are at great risk. Analysis based on studies in China reported contrasting relationship between spread rate and temperature, ranging from positive relationship reported for Wuhan, China (6), for Chinese cities with very low (<3°C) ambient temperatures (7), or positive (8) or negative (9) relationships for provinces across China, and lack of relationship between spread rates and temperature across cities in China (10). We believe that the conflicting nature of these results may derived, as shown for our analysis at the global scale prior to May 15, from confounding factors, including the spread across provinces in China as well as confinement policies. In contrast to these analyses for China, based on the analysis of single time periods and including provinces and cities with very low number of cases, where calculations involve significant uncertainty, we repeated our analysis across time period spanning 3 months, as well as an analysis based on the initial exponential spread, before confinement measures flattened the spread rate. Indeed, our analysis, including provinces across China, unveiled the risk of such spurious relationships, such as that obtained using global data until 15 March, 2020, due to confounding factors associated with the routes of spread of the epidemic. Similar partial evidence for a decrease in spread rates with increasing temperature for Barcelona. Spain (11), is likely to have also confounded the relationship between temperature and spread rates with the effects of confinement policies that reduced spread rates in that city as temperature increased during the study period. Hence, our findings at the global scale, and consolidated across multiple time periods following the pandemic state, reject the hypothesis of a relationship between COVID-19 spread rates and ambient temperature, consistent with those of recent studies that also reported no evidence for an association of epidemic growth with temperature using different approaches and data (12).

Many countries have employed strong lockdown and mandated school closures and restrictions of mass gatherings, social distancing to slow the growth of COVID-19 pandemic, which have successfully contained the spread on most cases. Our results suggest that release plans from confinement should not assume that spread rates will decrease with warm summer temperatures.

Data sources: The data on COVID-19 is available publicly across many sources; where downloadable data files are updated daily few are listed below;

*World health organization*(https://www.who.int/emergencies/diseases/novel-coronavirus-2019/situation-reports/).

*Johns Hopkins CSSE* (https://data.humdata.org/dataset/novel-coronavirus-2019-ncov-cases) [Accessed June 1, 2020].

*European Centre for Disease Prevention* and Control (https://www.ecdc.europa.eu/en/publications-data/download-todays-data-geographic-distribution-covid-19-cases-worldwide) (accessed June 1, 2020).

## Data Availability Statement

Publicly available datasets were analyzed in this study. This data can be found here: World health organization (https://www.who.int/emergencies/diseases/novel-coronavirus-2019/situation-reports/); Johns Hopkins CSSE (https://data.humdata.org/dataset/novel-coronavirus-2019-ncov-cases) (accessed March 25, 2020); European Centre for Disease Prevention and Control (https://www.ecdc.europa.eu/en/publications-data/download-todays-data-geographic-distribution-covid-19-cases-worldwide) (accessed March 26, 2020).

## Author Contributions

CD and TJ conceived, designed the research, and wrote the first draft. TJ conducted the analysis. All authors contributed to improving the paper and approved the submission.

## Conflict of Interest

The authors declare that the research was conducted in the absence of any commercial or financial relationships that could be construed as a potential conflict of interest.

## Supplementary Material

Figure S1 The apparent average (± SE) exponential rate of SARS-CoV-2 spread (γ), the average (and 95% confidence limits) of Effective Reproductive number of infection (Rt) and the average daily temperature (T_avg_) total case and number of days of the exponential growth period across nations and Chinese provinces where epidemics have been reported (data updated through May 31, 2020).

Figure S2 The relationship between the apparent exponential rate of SARS-CoV-2 spread (γ) and the Effective Reproductive number of infection (Rt) and the average daily temperature (T_avg_) across nations and Chinese provinces where > 100 cases of COVID-19 have been reported, as of Figure 1, but with data updated only until 15th March. The Gaussian function with temperature provided a significant fit for γ with temperature.

Figure S3 The relationship between the apparent exponential rate of SARS-CoV-2 spread (γ) and the Effective Reproductive number of infection (Rt) and the average daily temperature (T_avg_) across nations and Chinese provinces where > 100 cases of COVID-19 have been reported, as of Figure 1, but with all data updated only until 27th March.

Figure S4 The relationship between the apparent exponential rate of SARS-CoV-2 spread (γ) and the Effective Reproductive number of infection (Rt) and the average daily temperature (T_avg_) across nations and Chinese provinces, as of Figure 1, but with all data updated until 31st May.
