# Supplementary figures and images for: No Evidence for Temperature-Dependence of the COVID-19 Epidemic
Source: Front Public Health. 2020 Aug 26;8:436. doi: 10.3389/fpubh.2020.00436 (PMC7479095; doi:10.3389/fpubh.2020.00436)

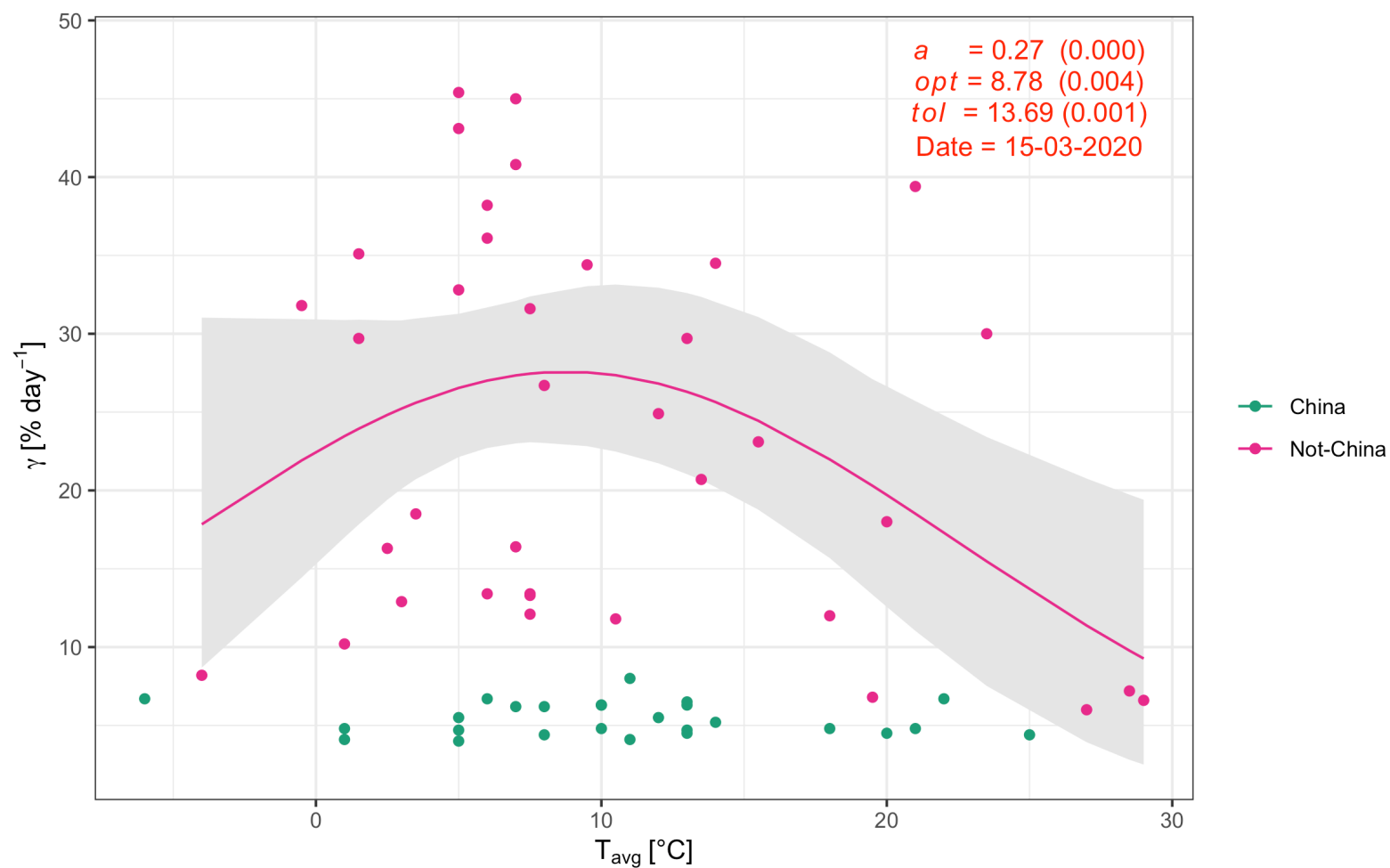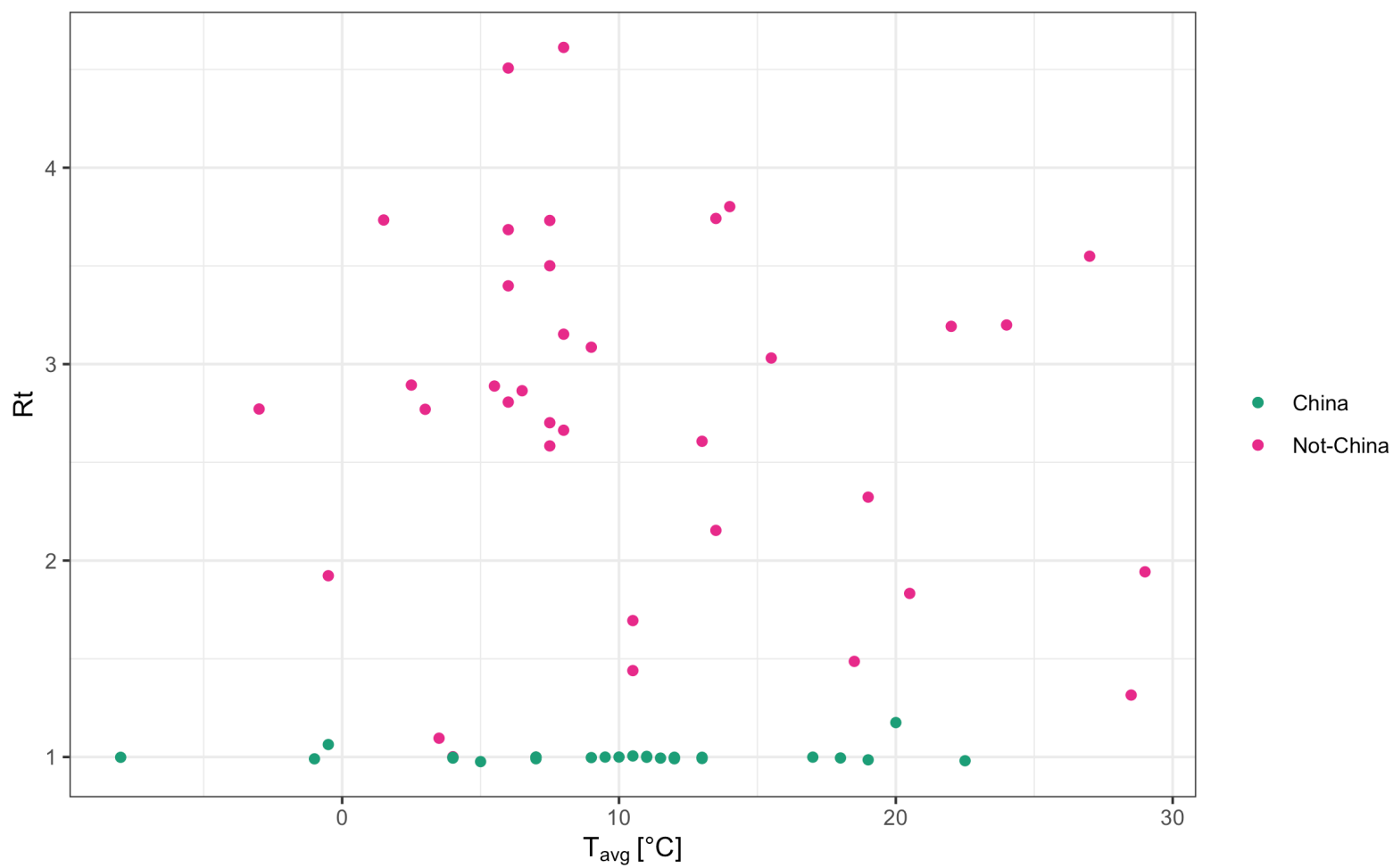

Supplement: Supplementary file 1 [file Data_Sheet_3.PDF]

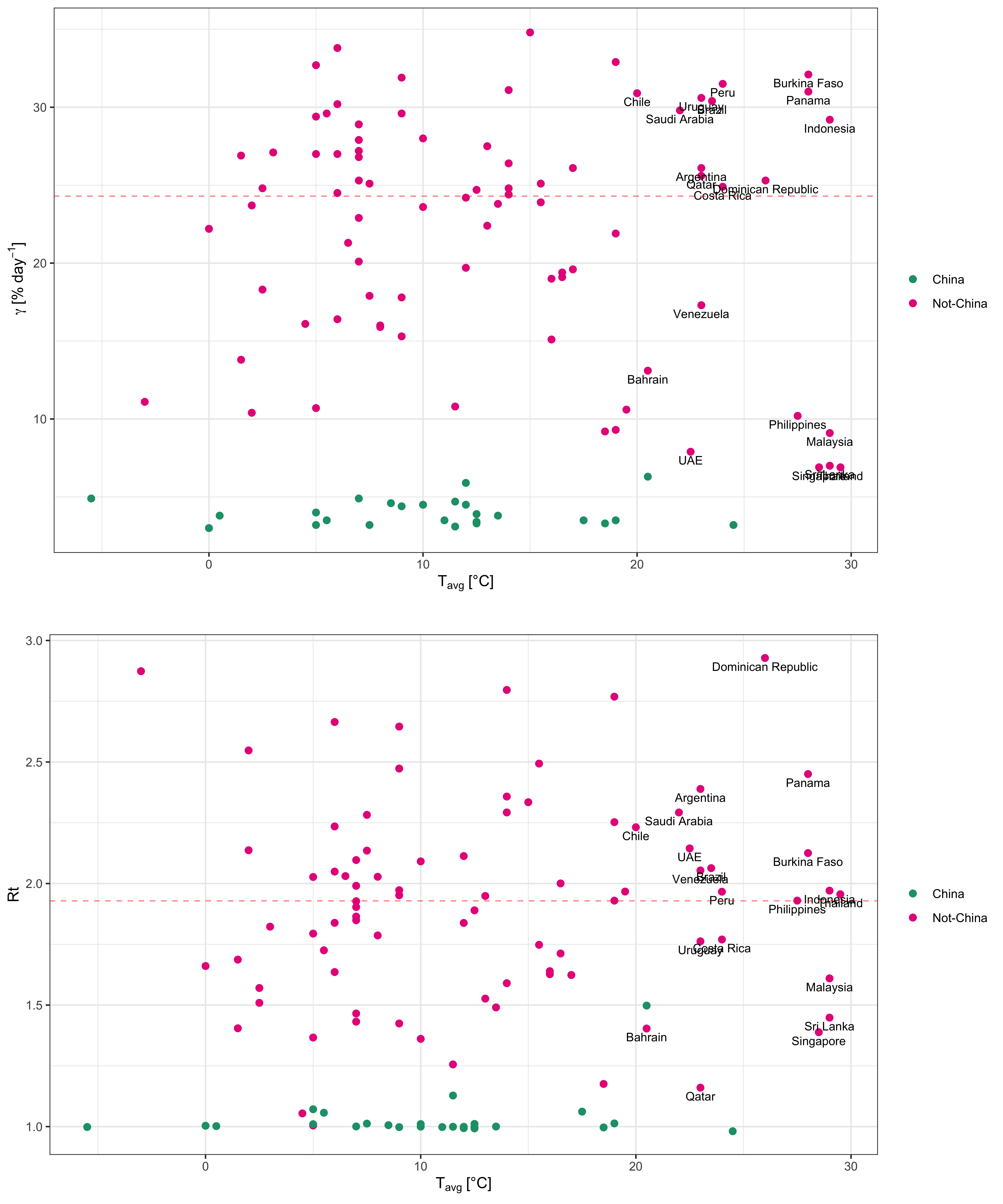

Supplement: Figure S1 — The apparent average (± SE) exponential rate of SARS-CoV-2 spread (γ), the average (and 95% confidence limits) of Effective Reproductive number of infection (Rt) and the average daily temperature (Tavg) total case and number of days of the exponential growth period across nations and Chinese provinces where epidemics have been reported (data updated through May 31, 2020). [file Image_1.PNG]

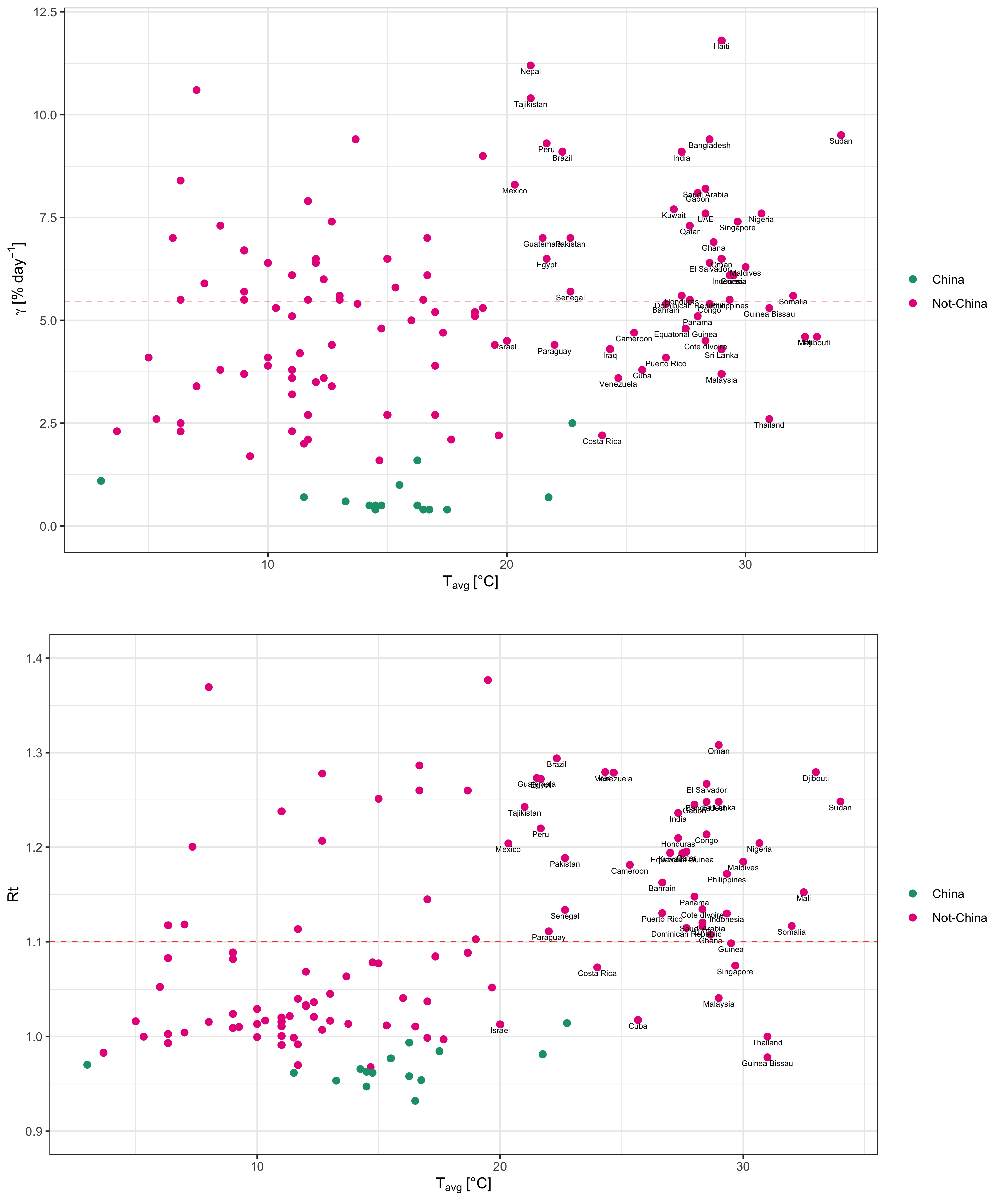

Supplement: Figure S2 — The relationship between the apparent exponential rate of SARS-CoV-2 spread (γ) and the Effective Reproductive number of infection (Rt) and the average daily temperature (Tavg) across nations and Chinese provinces where > 100 cases of COVID-19 have been reported, as of Figure 1, but with data updated only until 15th March. The Gaussian function with temperature provided a significant fit for γ with temperature. [file Image_2.PNG]

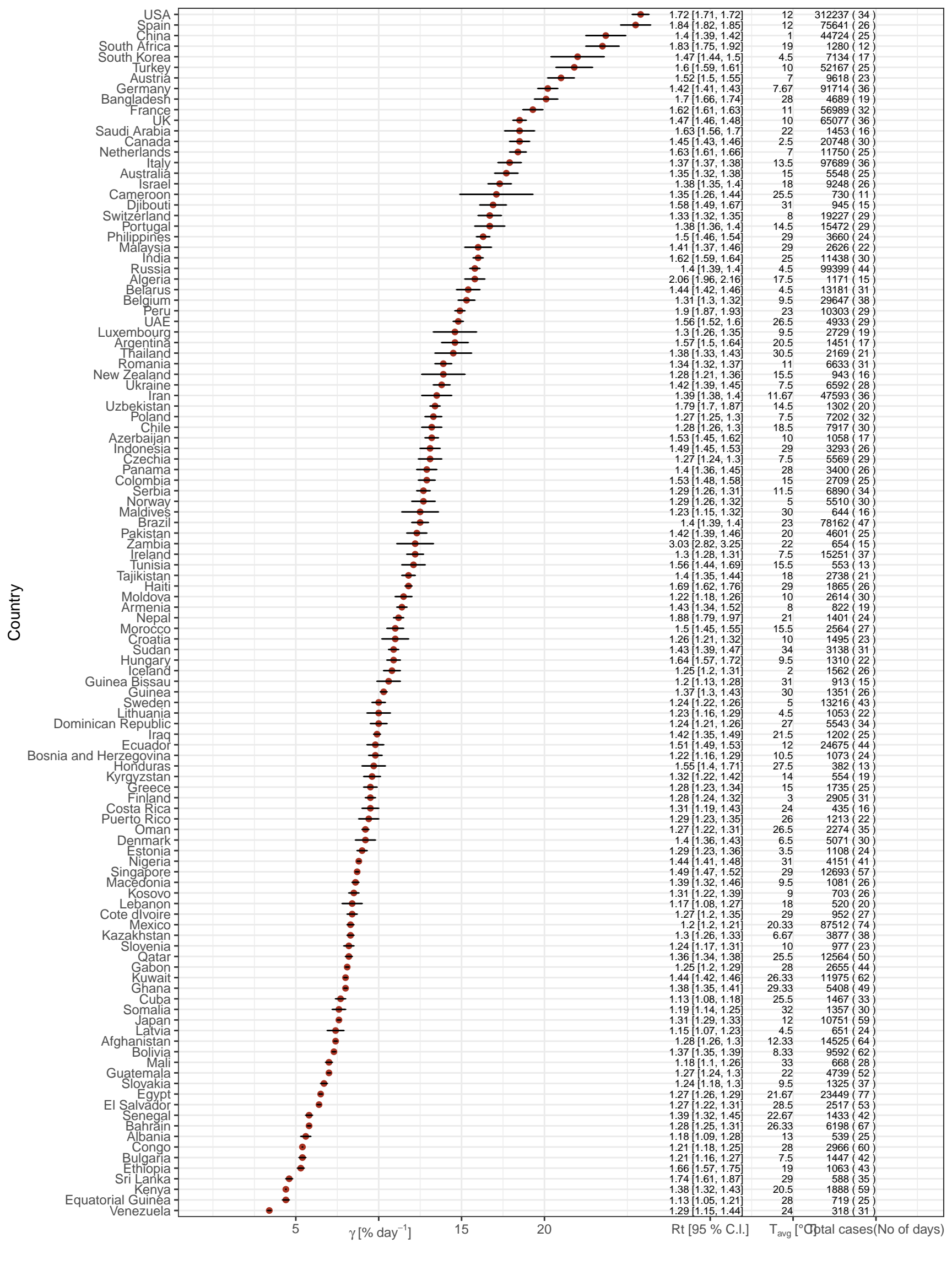

Supplement: Figure S3 — The relationship between the apparent exponential rate of SARS-CoV-2 spread (γ) and the Effective Reproductive number of infection (Rt) and the average daily temperature (Tavg) across nations and Chinese provinces where > 100 cases of COVID-19 have been reported, as of Figure 1, but with all data updated only until 27th March. [file Data_Sheet_1.PDF]

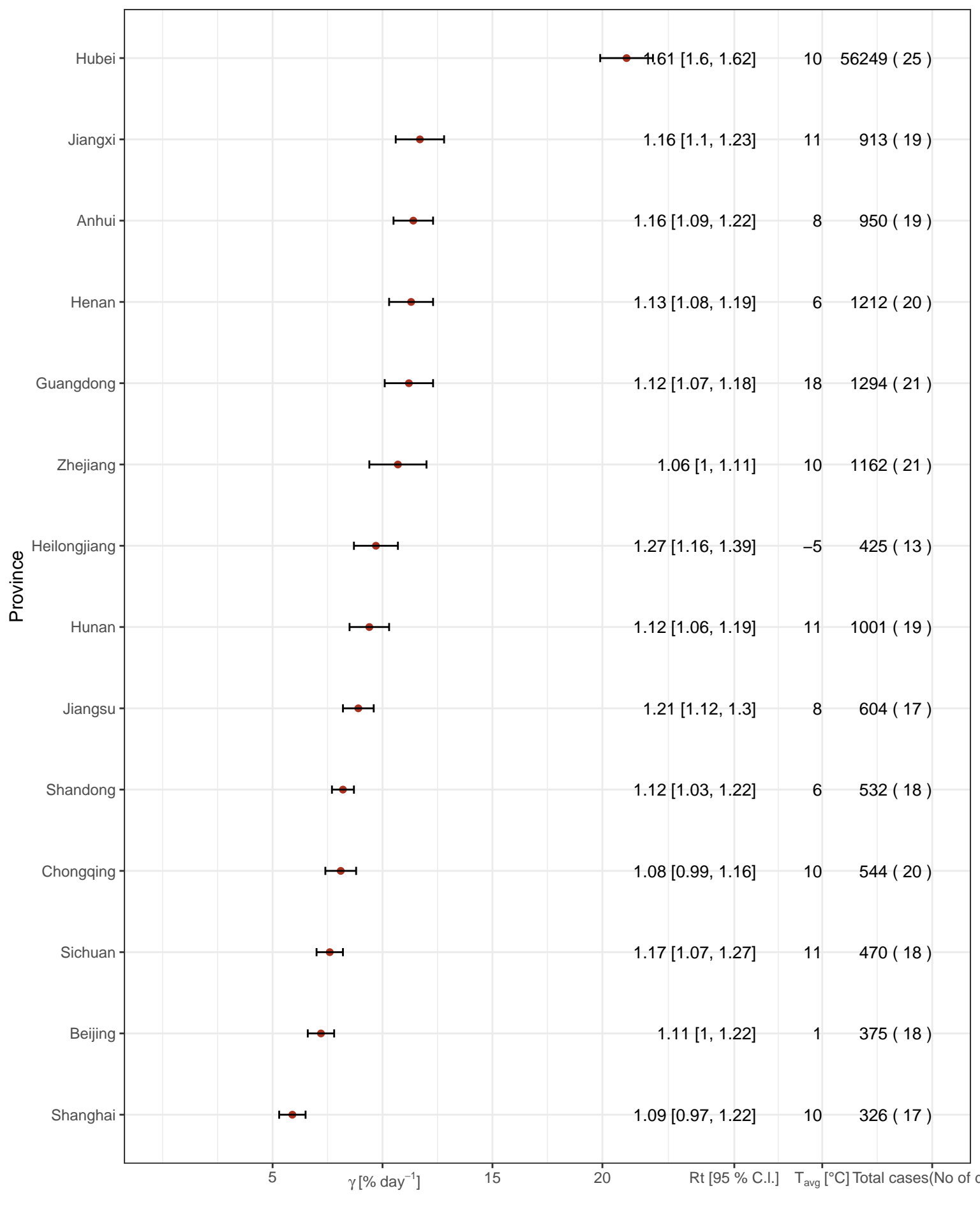

Supplement: Figure S4 — The relationship between the apparent exponential rate of SARS-CoV-2 spread (γ) and the Effective Reproductive number of infection (Rt) and the average daily temperature (Tavg) across nations and Chinese provinces, as of Figure 1, but with all data updated until 31st May. [file Data_Sheet_2.PDF]
